# Prostate-specific membrane antigen PET versus [^99m^Tc]Tc-MDP bone scan for diagnosing bone metastasis in prostate cancer: a head-to-head comparative meta-analysis

**DOI:** 10.3389/fmed.2024.1451565

**Published:** 2024-09-25

**Authors:** Yiming Wang, Yiran Qiu, Xingjian Yan

**Affiliations:** ^1^Department of Anesthesiology, First Hospital of Jilin University, Changchun, China; ^2^Department of Hand and Foot Surgery, Orthopedics Center, First Hospital of Jilin University, Changchun, China; ^3^Department of Urology Surgery, First Hospital of Jilin University, Changchun, China

**Keywords:** [^68^Ga]Ga-PSMA-11, [^18^F]DCFPyL, [^99m^Tc]Tc-MDP bone scan, prostate cancer, bone metastases

## Abstract

**Purpose:**

To evaluate the diagnostic performance of PSMA PET/CT, including [^68^Ga]Ga-PSMA-11 and [^18^F]DCFPyL, in comparison with the [^99m^Tc]Tc-MDP bone scan (BS) in identifying bone metastases among prostate cancer patients.

**Methods:**

A search was performed in the PubMed and Embase databases to locate pertinent publications from inception to February 12, 2024. The studies included were those that examined the diagnostic effectiveness of PSMA PET/CT (covering [^68^Ga]Ga-PSMA-11 and [^18^F]DCFPyL) compared to [^99m^Tc]Tc-MDP BS in identifying bone metastases among prostate cancer patients. The quality of the selected studies was evaluated using the Quality Assessment of Diagnostic Accuracy Studies-2 (QUADAS-2) checklist.

**Results:**

The meta-analysis included nine articles involving 702 patients. The sensitivity of PSMA PET/CT was higher compared to [^99m^Tc]Tc-MDP BS (0.98 vs. 0.85, *P* < 0.01), while the specificity of PSMA PET/CT was also higher than [^99m^Tc]Tc-MDP BS (0.97 vs. 0.70,*P* < 0.01). In subgroup analysis, the sensitivity of [^68^Ga]Ga-PSMA-11 PET/CT was higher compared to [^99m^Tc]Tc-MDP BS (0.98 vs. 0.86), while the specificity of [^68^Ga]Ga-PSMA-11 PET/CT was also higher than [^99m^Tc]Tc-MDP BS (0.98 vs. 0.65).

**Conclusion:**

Our meta-analysis demonstrates that PSMA PET/CT exhibits superior sensitivity and specificity in comparison with [^99m^Tc]Tc-MDP BS for identifying bone metastases in prostate cancer patients. Further research with head-to-head design is necessary to validate these results and evaluate the clinical effectiveness of these imaging methods.

**Systematic Review Registration:**

https://www.crd.york.ac.uk/prospero/, identifier PROSPERO CRD42024545112.

## Introduction

Prostate cancer (PCa) is a prevalent malignancy among men, with bone metastases being a common and serious complication in its advanced stages (1, 2). While lymph nodes are the most common site for PCa metastasis, bone is the second most common site, affecting around 70% of patients with advanced disease (3). These metastases often lead to significant morbidity, including pain, fractures, and decreased quality of life, underscoring the critical need for early and accurate diagnosis (4, 5). Early detection of bone metastases is crucial for optimizing treatment plans and enhancing patient outcomes (6).

Traditional methods for diagnosing bone metastases in PCa include computed tomography (CT), magnetic resonance imaging (MRI), and biopsy (7). CT and MRI provide detailed anatomical information but may lack the sensitivity to detect early or small metastatic lesions (8, 9). Although biopsies are considered definitive, they are invasive and not always practical for assessing multiple sites (10). Consequently, these conventional tools have limitations in sensitivity, specificity, and overall diagnostic accuracy, prompting the need for more advanced imaging techniques (11).

Recent advancements have introduced PSMA PET/CT, which employs radiotracers such as [^68^Ga]Ga-PSMA-11 and [^18^F]DCFPyL, as a cutting-edge method for detecting PCa bone metastases. In contrast, the [^99m^Tc]Tc-MDP BS remains a traditional and widely used technique in the diagnostic evaluation of these metastases (12). PSMA PET/CT is designed to target the PSMA protein, which is overexpressed in PCa cells, thereby providing a more targeted imaging approach (13). The radiotracers [^68^Ga]Ga-PSMA-11 and [^18^F]DCFPyL were approved by the FDA in December 2020 and May 2021, enhancing the specificity and effectiveness of PCa imaging (14). In contrast, [^99m^Tc]Tc-MDP BS, a longstanding method for detecting bone metastases, function by highlighting areas of increased bone turnover (15). Despite their widespread use, there is ongoing debate regarding the diagnostic superiority of PSMA PET/CT compared to [^99m^Tc]Tc-MDP BS, with conflicting evidence on their relative sensitivities and specificities (16).

This meta-analysis aims to conduct a head-to-head comparative evaluation of PSMA PET/CT (including [^68^Ga]Ga-PSMA-11 and [^18^F]DCFPyL) and [^99m^Tc]Tc-MDP BS in diagnosing bone metastases about PCa.

## Materials and methods

The meta-analysis adhered to the guidelines set by the Preferred Reporting Items for a Systematic Review and Meta-analysis of Diagnostic Test Accuracy (PRISMA-DTA) (17). The protocol has been registered with PROSPERO under the registration number CRD42024545112. Additionally, we have provided the PRISMA checklist as Supplementary Table 1, which is now referenced in the manuscript.

### Search strategy

A thorough search was performed in the PubMed and Embase databases to locate available publications from 2006 to February 12, 2024. The search used the keywords: (“Prostatic Neoplasms” OR “Prostatic Cancers” OR “Prostatic Cancer” OR “Prostate Cancers” OR “Prostate Cancer” OR “Prostatic Neoplasm” OR “Prostate Neoplasm” OR “Prostate Neoplasms” OR “Prostate tumor” OR “prostatic tumor”) AND (“Positron Emission Tomography Computed Tomography” OR “positron emission tomography/computed tomography” OR “PET/CT”) AND (“Bone scan” OR “Bone scintigraphy”) AND (“Bone metastasis” OR “Bone metastases”). No language or other filters were applied during the search. Supplementary Table 2 provides more details. Additionally, the reference lists of the included studies were manually reviewed to uncover further relevant studies. The search and study selection process was conducted independently by two reviewers. One reviewer identified seven of the nine included studies, while the other identified all nine, resulting in an overlap rate of approximately 78%. Discrepancies were resolved through discussion or by consulting a third reviewer to ensure accurate aggregation and inclusion of studies.

### Inclusion and exclusion criteria

The inclusion criteria for our study were defined as follows: (1) Population (P): Individuals diagnosed with PCa; (2) Intervention (I): Utilization of PSMA PET/CT ([^68^Ga]Ga-PSMA-11 and [^18^F]DCFPyL) for the assessment of bone metastasis; (3) Comparator (C): Employment of [^99m^Tc]Tc-MDP BS for the evaluation of bone metastasis; (4) Outcomes (O): The primary outcomes were sensitivity and specificity in patient-based analysis; (5) Study design (S): Both retrospective and prospective studies were included in the analysis.

Duplicated studies, case reports, abstracts, letters, reviews, meta-analyses, clearly irrelevant titles and abstracts, no head-to-head comparison and data not available were excluded.

### Quality assessment

The quality of the included articles was assessed by two researchers independently utilizing the Quality Assessment of Diagnostic Accuracy Studies-2 (QUADAS-2) tool (18). This tool covers four domains: (1) patient selection, (2) index test, (3) reference standard, and (4) flow and timing. The risk of bias for each domain was classified as “high risk”, “low risk”, or “unclear risk”. For risk of bias, patient selection was rated as low risk if consecutive patients were included, high risk if there were inappropriate exclusions, and unclear if not specified. The index test was rated as low risk if the cut-off value was pre-specified, high risk if determined post hoc, and unclear if not reported. The reference standard was rated as low risk if diagnosed by two or more physicians or if pathology plus imaging was used, high risk if only one physician, and unclear if not reported. Flow and timing was rated as low risk if the time interval was less than three months, high risk if more than three months, and unclear if not described. For applicability concerns, patient selection was rated as high risk if the study population differed from our meta-analysis criteria, low risk if consistent, and unclear if not specified, while the index test and reference standard were both rated as low risk if consistent with our meta-analysis definitions. Detail information was provided in the Supplementary Table 3.

### Data extraction

Two researchers independently conducted data extraction for all included papers. The extracted data covered the following categories: (1) author and year of publication, (2) radiotracer used, (3) study characteristics, including country, study design, reference standard, and study period, and (4) patient characteristics, including the number of patients, PSA level, mean or median age, Gleason score, and clinical indication. For the extraction of true positive (TP), true negative (TN), false positive (FP), and false negative (FN) values, if these were not directly provided in the articles, we utilized the calculator tool in RevMan 5.4 to back-calculate these values based on reported sensitivity and specificity.

In instances of disagreement, the researchers deliberated on the matter until they reached a consensus, ensuring the accuracy of the extracted data.

### Statistical analysis

The bivariate random-effects model was employed to jointly assess sensitivity and specificity, providing a more accurate estimation of diagnostic performance. This method accounts for the correlation between sensitivity and specificity across studies. Confidence intervals were calculated based on the bivariate model, ensuring robust and reliable results. The degree of heterogeneity within and between groups was evaluated using the Cochrane Q and I^2^ statistics (19). Significant heterogeneity, defined as *P* < 0.05 or I^2^ > 50%, prompted further meta-regression and leave-one-out sensitivity analysis to identify its source.

Publication bias was assessed utilizing funnel plot analysis and Egger’s test (20). A *P*-value of less than 0.05 was deemed statistically significant for all tests. All statistical analyses were conducted using R software version 4.3.3 and Stata 15.1.

## Results

### Study selection

**FIGURE 1:**
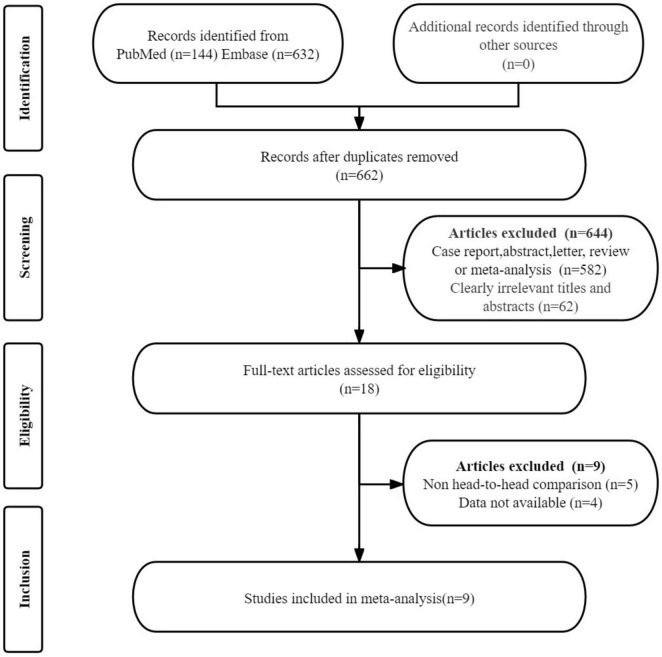
PRISMA flow diagram illustrating the study selection process.

The initial search yielded 776 publications. After removing 114 duplicate studies, 644 studies were excluded on the basis that they did not meet the eligibility criteria. A detailed review of the full texts of the remaining 18 articles led to the exclusion of an additional nine studies: four studies were excluded due to the unavailability of data (TP, FP, FN, and TN), and five studies were excluded because they were not head-to-head comparison articles. Ultimately, nine articles that evaluated the diagnostic efficacy of PSMA PET/CT (including [^68^Ga]Ga-PSMA-11 and [^18^F]DCFPyL) and [^99m^Tc]Tc-MDP BS were included in the meta-analysis (21–29). The article selection process is illustrated using the PRISMA flow diagram shown in Figure 1.

### Study description and quality assessment

The nine eligible studies included 702 PCa patients (range from 28 to 138). Of these studies, eight were retrospective studies (21–23, 25–29), while one article was prospective study (24). Six articles used best valuable comparator (BVC) as the reference standard (22–25, 27, 29), while three relied on imaging follow-up (21, 26, 28). Regarding the radiotracer of PSMA PET/CT, 7 articles used [^68^Ga]Ga-PSMA-11 (21, 22, 24–28),while the remaining two articles used [^18^F]DCFPyL (23, 29). Among the included studies, four utilized planar BS (21, 22, 25, 27), three employed single photon emission computed tomography (SPECT) imaging (23, 24, 29), and two studies incorporated both planar BS and SPECT imaging (26, 28). A summary of the study and patient characteristics of the included studies is presented in Table 1.

**TABLE 1:** Study and patient characteristics of the included studies.

References	Radiotracer	Study characteristics					Patient characteristics				
		Country	Study design	Reference standard	Period	Analysis	Number of patients	PSA level (ng/ml)	Age (year)	Gleason Score	Clinical indication
Hu et al. (23)	18F-DCFPyL	China	Retro	BVC	2020–2022	PB	31	70.88 ± 28.6 (0.15–372.08)	Mean ± SD: 67.83 ± 6.65	≤ 6 (*n* = 7) = 7 (*n* = 8) ≥ 8 (*n* = 16)	Initial staging (*n* = 31)
Wilson et al. (29)	18F-DCFPyL	USA	Retro	BVC	2021–2022	PB	91	5.4 (1.85–16.45)	Median (range):69 (63–75)	≤ 6 (*n* = 2) 7 (*n* = 25) ≥ 8 (*n* = 61)	NA
Caglar et al. (22)	68Ga-PSMA-11	Turkey	Retro	BVC	2014–2019	PB	95	21.6 (0.22–1465)	Mean (range):69 (43–90)	NA	Initial staging (*n* = 31); BCR (*n* = 27); mCRPC (*n* = 37)
Simsek et al. (26)	68Ga-PSMA-11	Turkey	Retro	Imaging follow-up	2015–2019	PB	138	18.3 (0.3–853)	66 (49–92)	≤ 6 (*n* = 14) 7 (*n* = 37) ≥ 8 (*n* = 87)	Initial staging (*n* = 77); BCR (*n* = 61)
Soydal et al. (27)	68Ga-PSMA-11	Turkey	Retro	BVC	2014–2018	PB	46	11.6 (1.0–1658)	68.4 ± 6.4 (51–81)	≤ 6 (*n* = 8) 7 (*n* = 13) ≥ 8 (*n* = 22)	Initial staging (*n* = 25); BCR (*n* = 11); mCRPC (*n* = 10)
Uslu-Beşli et al. (28)	68Ga-PSMA-11	Turkey	Retro	Imaging follow-up	2015–2016	PB	28	25.49 ± 32.7 (0.5–125.1)	67.3 ± 7.4	≤ 6 (*n* = 3) 7 (*n* = 14) ≥ 8 (*n* = 11)	Initial staging and restaging (*n* = 28)
Acar et al. (21)	68Ga-PSMA-11	Turkey	Retro	Imaging follow-up	2015–2017	PB	34	51 ± 159 (0–912)	66 ± 9.5 (50–88)	Mean: 8 (6–9)	Initial staging and restaging (*n* = 34)
Lengana et al. (24)	68Ga-PSMA-11	South Africa	Pro	BVC	NA	PB	113	18.3 (0.3–853)	66.65 ± 7.89 (43–88)	≤ 6 (*n* = 10) 7 (*n* = 42) ≥ 8 (*n* = 61)	Initial staging (*n* = 113)
Pyka et al. (25)	68Ga-PSMA-11	Germany	Retro	BVC	2012–2015	PB	126	NA	68.9 ± 7.7 (49–89)	NA	Initial staging (*n* = 37); BCR (*n* = 49); mCRPC (*n* = 40)

Pro prospective; Retro retrospective; BS bone scintigraphy; PB patient-based; BVC best valuable comparator.

**FIGURE 2:**
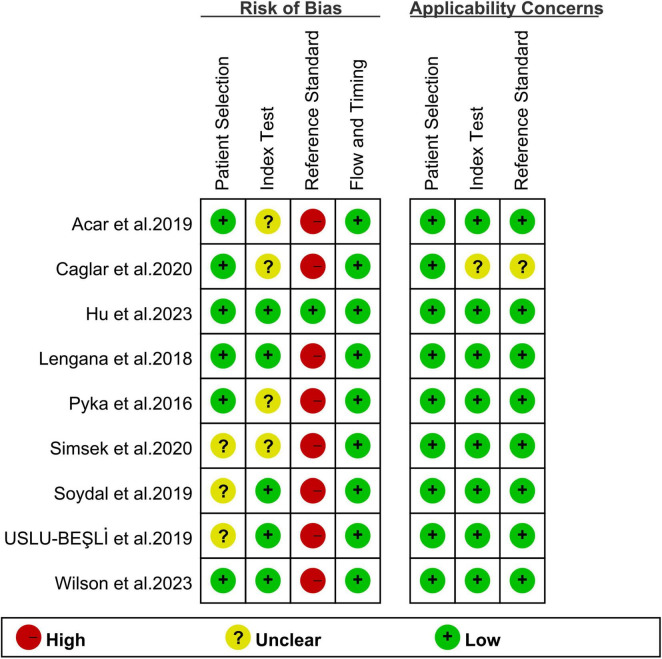
Risk of bias and applicability concerns of the included studies using the Quality Assessment of Diagnostic Performance Studies QUADAS-2 tool.

The risk of bias for each study, as assessed utilizing the QUADAS-2 tool, is shown in Figure 2. For patient selection, three studies were rated as “unclear risk” due to a lack of information on whether consecutive patients were included (26–28). With regard to the index test, four studies were assigned an “unclear risk” rating due to a lack of information regarding the pre-determined cut-off values applied (21, 22, 25, 26). In terms of the reference standard, eight studies were rated as having an “unclear risk” due to the fact that the final diagnosis was not independently determined by two or more physicians (21, 22, 24–29). Overall, the quality assessment revealed no major concerns about the quality of the included studies.

### Comparing the sensitivity and specificity of PSMA PET/CT to [^99m^Tc]Tc-MDP BS for detecting bone metastases in PCa

**FIGURE 3:**
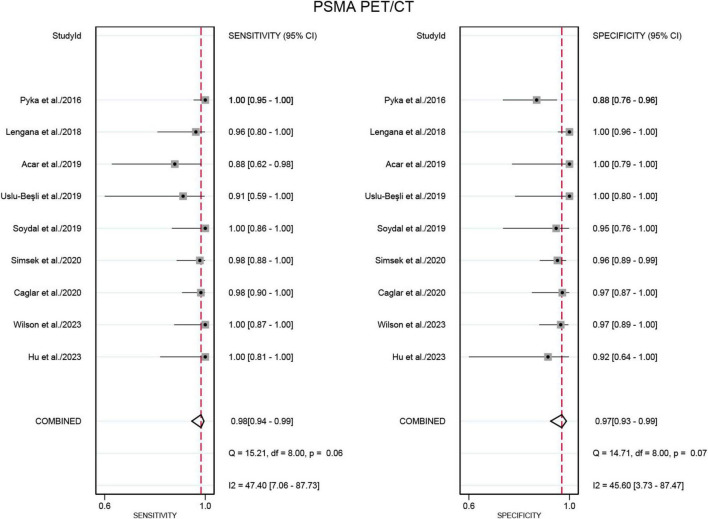
Forest plot for the sensitivity and specificity of PSMA PET/CT in detecting bone metastases in prostate cancer. The plot displays individual study estimates (squares) with corresponding 95% confidence intervals (horizontal lines) and the pooled sensitivity estimate (diamond) for both modalities. The size of the squares represents the relative weight of each study in the meta-analysis.

**FIGURE 4:**
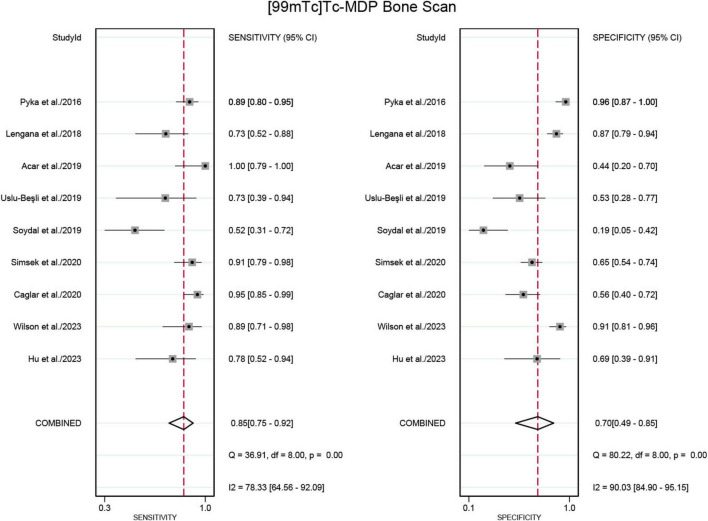
Forest plot for sensitivity and specificity of [^99m^Tc]Tc-MDP BS in detecting bone metastases in prostate cancer. The plot displays individual study estimates (squares) with corresponding 95% confidence intervals (horizontal lines) and the pooled sensitivity estimate (diamond) for both modalities. The size of the squares represents the relative weight of each study in the meta-analysis.

Nine studies were included in the analysis (21–29). The sensitivity for detecting bone metastases in PCa in patient-based analysis was significantly higher for PSMA PET/CT at 0.98 (95% CI: 0.94–0.99) compared to [^99m^Tc]Tc-MDP BS, which had a sensitivity of 0.85 (95% CI: 0.75–0.92) (Figures 3, 4). This difference in sensitivity between PSMA PET/CT and [^99m^Tc]Tc-MDP BS was statistically significant (*P* < 0.01). Similarly, the specificity of PSMA PET/CT was 0.97 (95% CI: 0.93–0.99), markedly higher than the specificity of [^99m^Tc]Tc-MDP BS at 0.70 (95% CI: 0.49–0.85) (Figures 3, 4). This difference in specificity was also statistically significant (*P* < 0.01).

The sensitivity and specificity of [^99m^Tc]Tc-MDP BS exhibited I^2^ values of 78.33% and 90.03%,respectively. The meta-regression analysis for sensitivity revealed no source of heterogeneity (Table 2). However, a leave-one-out sensitivity analysis revealed that omitting the study by Soydal et al. resulted in a reduction of the I^2^ to 51%, indicating that this study may be a significant source of heterogeneity (Supplementary Figure 1) (27). With regard to specificity, the leave-one-out sensitivity analysis identified no source of heterogeneity (Supplementary Figure 2).

**TABLE 2:** Subgroup analysis and meta-regression analysis for [^99m^Tc]Tc-MDP bone scan.

Covariate	Studies, *n*	Sensitivity (95%CI)	*P*-value	Specificity (95%CI)	*P*-value
No. of patients			0.85		0.09
≤ 100	6	0.85 (0.68–0.96)		0.57 (0.35–0.78)	
> 100	3	0.85 (0.75–0.93)		0.84 (0.63–0.98)	
Region			0.49		0.10
Europe	6	0.87 (0.72–0.97)		0.58 (0.34–0.81)	
Non-Europe	3	0.81 (0.69–0.90)		0.88 (0.82–0.93)	
Study design			0.40		0.34
Retrospective	8	0.87 (0.75–0.95)		0.65 (0.44–0.83)	
Prospective	1	0.85 (0.75–0.93)		0.87 (0.79–0.94)	
Reference standard			0.34		0.28
BVC	6	0.82 (0.68–0.92)		0.74 (0.48–0.93)	
Imaging follow-up	3	0.92 (0.74–1.00)		0.58 (0.45–0.71)	

BVC best valuable comparator.

### Comparing the sensitivity and specificity of [^68^Ga]Ga-PSMA-11 PET/CT to [^99m^Tc]Tc-MDP BS for detecting bone metastases in PCa

**FIGURE 5:**
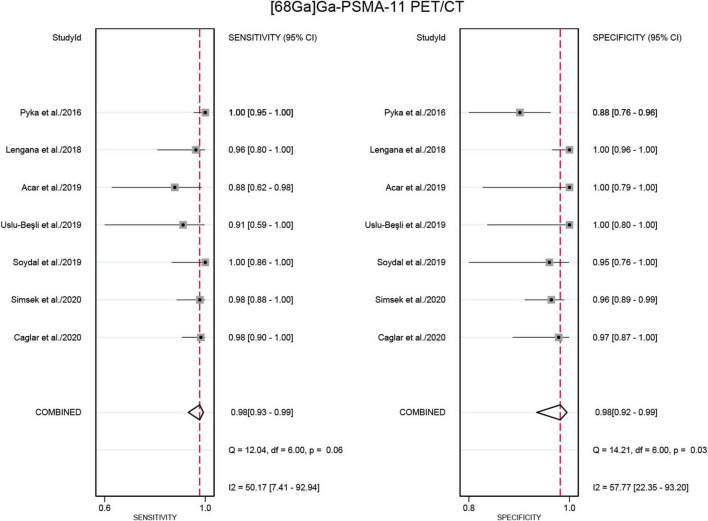
Forest plot for the sensitivity and specificity of [^68^Ga]Ga-PSMA-11 PET/CT in detecting bone metastases in prostate cancer. The plot displays individual study estimates (squares) with corresponding 95% confidence intervals (horizontal lines) and the pooled sensitivity estimate (diamond) for both modalities. The size of the squares represents the relative weight of each study in the meta-analysis.

**FIGURE 6:**
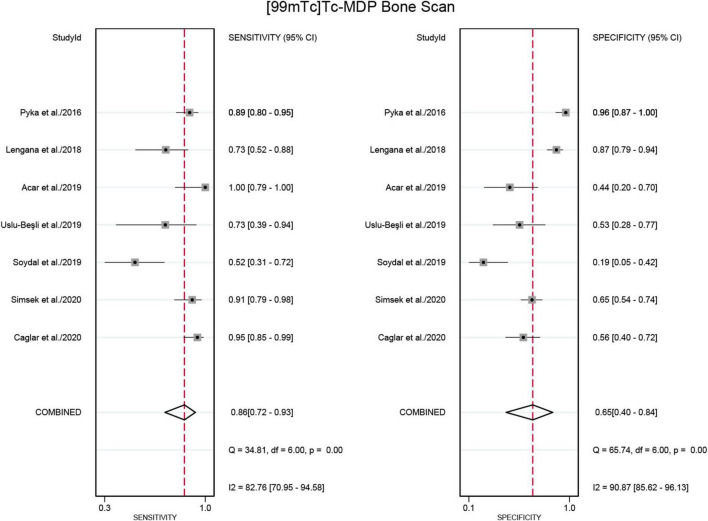
Forest plot showing the sensitivity and specificity of [^99m^Tc]Tc-MDP BS in detecting bone metastases in prostate cancer (including only studies that used [^68^Ga]Ga-PSMA-11 PET/CT for comparison). The plot displays individual study estimates (squares) with corresponding 95% confidence intervals (horizontal lines) and the pooled specificity estimate (diamond) for both modalities. The size of the squares represents the relative weight of each study in the meta-analysis.

Seven studies were included in the analysis (21, 22, 24–28). The pooled sensitivity for detecting bone metastases in PCa in patient-based analysis was 0.98 (95% CI: 0.93–0.99) for [^68^Ga]Ga-PSMA-11 PET/CT, compared to 0.86 (95% CI: 0.72–0.93) for [^99m^Tc]Tc-MDP BS, with this difference in sensitivity being statistically significant (P < 0.01) (Figures 5, 6). Additionally, the pooled specificity was 0.98 (95% CI: 0.92–0.99) for [^68^Ga]Ga-PSMA-11 PET/CT, significantly higher than the specificity of 0.65 (95% CI: 0.40–0.84) for [^99m^Tc]Tc-MDP BS (*P* < 0.01) (Figures 5, 6).

### The rates of therapeutic management changes after PSMA PET/CT

**FIGURE 7:**
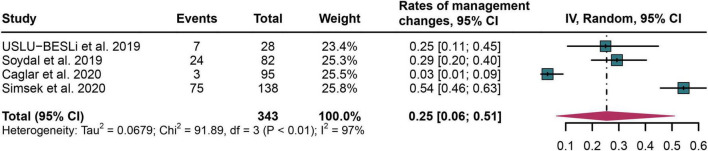
Forest plot of therapeutic management change rates following PSMA PET/CT in prostate cancer bone metastasis.

Four studies were included in the analysis (22, 26–28). The pooled rates of management changes in PCa cancer patients after PSMA PET/CT was 0.25 (95% CI: 0.06–0.51) (Figure 7).

### Publication bias

The funnel plot asymmetry test revealed no evidence of significant publication bias for any of the outcomes (Egger’s test: all *P* > 0.05) (Supplementary Figures 3–6).

## Discussion

A number of studies have recently demonstrated that both PSMA PET/CT and [^99m^Tc]Tc-MDP BS possess strong diagnostic capabilities for detecting bone metastasis in PCa (8, 16, 23). However, a systematic comparison between these two diagnostic tools to determine which has superior diagnostic accuracy remains uncertain (30). The head-to-head comparative meta-analysis presented here aims to address this gap. Our analysis indicates that PSMA PET/CT, with its higher sensitivity and specificity, shows greater promise in identifying bone metastasis in PCa compared to [^99m^Tc]Tc-MDP BS. The data suggests that PSMA PET/CT could potentially replace [^99m^Tc]Tc-MDP BS as the preferred diagnostic method, provided its accessibility and cost-effectiveness are addressed.

The main findings of this meta-analysis reveal that PSMA PET/CT significantly outperforms [^99m^Tc]Tc-MDP BS in diagnosing bone metastasis in PCa. Specifically, PSMA PET/CT demonstrated higher sensitivity (0.98 vs. 0.85) and specificity (0.97 vs. 0.70) in comparison to ^99m^Tc-MDP BS. Subgroup analysis further showed that ^68^Ga-PSMA-11 PET/CT had higher sensitivity (0.98 vs. 0.86) and specificity (0.98 vs. 0.65) than [^99m^Tc]Tc-MDP BS. The enhanced sensitivity and specificity of PSMA PET/CT are attributed to its ability to target the PSMA protein, which is highly expressed on PCa cells, allowing for more accurate detection of metastatic sites (31, 32). This high affinity and specific binding result in clearer imaging and better differentiation between malignant and benign lesions (33). Overall, these findings suggest that PSMA PET/CT, particularly with [^68^Ga]Ga-PSMA-11, provides a more reliable diagnostic tool for detecting bone metastasis in PCa, potentially offering significant improvements in patient management and treatment planning.

Comparing our study with previous meta-analyses, we provide significant advancements and address the limitations noted in earlier researches. Ji et al. (16) conducted the first systematic evaluation of [^68^Ga]Ga-PSMA-11 PET/CT versus [^99m^Tc]Tc-MDP BS for diagnosing bone metastasis in PCa (16). Their results indicated higher sensitivity (98% vs. 83%) and specificity (97% vs. 61%) for [^68^Ga]Ga-PSMA-11 PET/CT. However, the study had limitations, including a small number of included articles (only six) and the absence of standardized statistical tests to compare sensitivity and specificity between the diagnostic tools. Additionally, the diagnostic performance of the key radiotracer [^18^F]DCFPyL was not fully explored, indicating a need for further investigation in this area.

In a comprehensive meta-analysis, Chow et al. (34) compared PSMA PET/CT with conventional tools, including BS and MRI, for initial PCa diagnosis, lymph node metastasis, and bone metastasis (34). Despite its thorough approach, the study has limitations such as not employing leave-one-out sensitivity analyses or meta-regression analysis to explore the sources of high heterogeneity in the PET vs. BS comparison subgroup. Furthermore, our study incorporated a larger pool of literature and included the analysis of [^18^F]DCFPyL, enhancing the robustness and comprehensiveness of our findings. We performed subgroup analyses based on different PET/CT radiotracers, providing a detailed comparison of diagnostic performance between specific radiotracers and BS. This methodological rigor allowed us to deliver more precise insights into the diagnostic performance of various PSMA PET/CT radiotracers in comparison to BS.

In the other meta-analysis, Shen et al. (35) compared choline-PET/CT, MRI, SPECT, and BS in the diagnosis of bone metastases in patients with PCa and concluded that SPECT imaging has demonstrated its potential to significantly improve diagnostic accuracy compared to traditional conventional imaging techniques including planar BS. The current study is constrained by the limited number of available SPECT studies, which restricts our ability to conduct a comprehensive comparison between PSMA PET/CT and SPECT. This limitation highlights the need for future research that rigorously compares PSMA PET with SPECT, a topic that holds considerable potential for advancing diagnostic methodologies.

In our meta-analysis, we chose to exclude studies utilizing [^18^F]PSMA-1007 as a radiotracer. This decision was primarily driven by the higher rate of false-positive findings associated with [^18^F]PSMA-1007, particularly in bone imaging (36). Furthermore, there is a notable lack of studies that directly compare [^18^F]PSMA-1007 PET imaging with conventional BS in a head-to-head manner, limiting our ability to perform a comprehensive meta-analysis on this particular radiotracer. However, [^18^F]PSMA-1007 remains a significant PSMA radiotracer, widely used in clinical practice due to its high sensitivity in detecting PCa lesions. Future research should focus on better understanding its diagnostic performance, particularly in comparison to other imaging modalities.

Since PSMA PET/CT exhibits higher sensitivity and specificity in comparison with [^99m^Tc]Tc-MDP BS in detecting bone metastasis in PCa, making it a seemingly better choice when only diagnostic performance is considered. The advantages of PSMA PET/CT include its superior diagnostic accuracy and ability to provide more detailed imaging, which is crucial for early detection and treatment planning (37, 38). However, PSMA PET/CT is less widely available and typically more expensive than BS, which might limit its accessibility for some patients (39). Moreover, although both imaging modalities are generally safe, the choice between them should be guided by the patient’s individual condition and the specific clinical context (40). A combined diagnostic model utilizing both PSMA PET/CT and BS could potentially enhance diagnostic performance by leveraging the complementary strengths of each modality (41). Clinicians should carefully evaluate the benefits and limitations of each tool to make informed decisions tailored to patient-specific circumstances (42).

Interpreting the findings of this meta-analysis, several limitations should be considered. Firstly, the heterogeneity among the included studies might have influenced the pooled sensitivities and specificities of PSMA PET/CT and [^99m^Tc]Tc-MDP BS. To address this, we performed leave-one-out sensitivity analyses, which identified Soydal et al. (27) as a potential source of heterogeneity. Secondly, the number of studies in the head-to-head comparison of [^18^F]DCFPyL PET/CT versus [^99m^Tc]Tc-MDP BS was relatively small, indicating the need for well-designed prospective head-to-head studies to confirm our findings. Thirdly, not all patients underwent pathological biopsy as the gold standard, which may introduce bias into the results. Future research should include more studies where pathological biopsy is used as the gold standard.

## Conclusion

Based on our findings, the meta-analysis illustrates that PSMA PET/CT (including [^68^Ga]Ga-PSMA-11 and [^18^F]DCFPyL) demonstrates higher sensitivity and comparable specificity to [^99m^Tc]Tc-MDP BS in the identification of bone metastases in PCa patients. Additional research with head-to-head design and extensive pathological data is necessary to validate these observations and to assess the clinical utility of these imaging techniques.

## Data Availability

The original contributions presented in the study are included in the article/Supplementary material, further inquiries can be directed to the corresponding author.
